# Unsafe firearm storage among family caregivers of older persons living with dementia

**DOI:** 10.1371/journal.pone.0360412

**Published:** 2026-10-28

**Authors:** Jiaming Liang, Alexander Testa

**Affiliations:** University of Texas Health Science Center at Houston, School of Public Health, San Antonio, TX, United States of America; Kennesaw State University, UNITED STATES OF AMERICA

## Abstract

**Purpose:**

Firearm access in dementia caregiving households poses safety risks. This study examines household firearm ownership and storage practices among dementia caregivers, identifying factors associated with unsafe firearm storage.

**Methods:**

We analyzed pooled data from 2017, 2021, 2022, and 2023 Behavioral Risk Factor Surveillance System (BRFSS). Weighted logistic regression assessed associations between caregiver characteristics and firearm storage behaviors (unloaded vs. loaded, locked vs. unlocked).

**Results:**

Dementia caregivers were more likely to live in firearm-owning households than non-caregivers (40.57% vs. 35.12%). Among caregivers in firearm-owning households, 35.8% reported loaded storage, and 50.8% of those also reported unlocked storage. Older caregivers are more likely to report unlocked firearm storage (50–64 years: OR=3.74, 95%CI: 1.75–8.01; 65 + years: OR=5.39, 95%CI: 2.02–14.40; reference: < 50 years). Male caregivers had higher odds of reporting a loaded firearm in the household (OR=1.98, 95% CI: 1.32–2.96). In contrast, longer caregiving duration (>5 years: OR=0.36, 95%CI: 0.16–0.78) and assisting with household tasks (OR=0.27, 95%CI: 0.11–0.63) were associated with safer storage, though this protective effect was only observed among younger caregivers.

**Conclusion:**

Household firearm storage patterns among dementia caregivers varied by caregiver age, sex, and caregiving context, highlighting the need for tailored firearm-safety counseling and caregiver-focused public health strategies.

## Introduction

The United States has the highest rate of firearm ownership globally [1], with an estimated 300–400 million firearms in circulation [2,3] and approximately 40% of households reporting the presence of a firearm [4]. While firearm ownership is a constitutionally protected right in the U.S., secure firearm storage, defined as storing firearms unloaded and locked, is critical for reducing the risk of firearm-related injury and morbidity [5–8]. To be sure, research demonstrates that secure firearm storage is associated with lower rates of unintentional firearm injuries and firearm-related deaths [6–8], and may be particularly beneficial in households with vulnerable populations such as individuals living with Alzheimer’s disease and related dementias (ADRD), especially considering findings highlighting ADRD elevates the risk for self-directed harm [9–11] and interpersonal aggression [12].

ADRD is a progressive neurodegenerative condition characterized by cognitive decline, memory impairment, and behavioral changes [13]. As the disease advances, individuals with dementia may experience agitation, confusion, and impaired judgment, potentially increasing the risk of firearm-related harm, including self-inflicted injuries, accidental shootings, and violence toward household members when firearms are left unsecured and easily accessible. Dementia caregivers often provide assistance with medical needs, household tasks, personal care, and safety [14]. When firearms are present, caregivers may also encounter decisions related to firearm storage and access, although their role in such decisions depends on living arrangements, firearm ownership, and access [15]. Given the increasing prevalence of dementia—which currently affects 6 million Americans and is projected to reach nearly 14 million by 2060 [16]—understanding firearm storage and access in the context of dementia caregiving is an urgent public health concern. Even so, research on how caregiver characteristics are associated with firearm storage behaviors remains nascent.

While existing studies have examined firearm-related risks associated with dementia [15,17], few have investigated how features of caregivers themselves—particularly the demands and stressors placed on caregivers—may shape firearm storage behaviors. Moreover, little is known about how demographic, health-related correlates of firearm storage [18–21], and caregiving characteristics interact to influence firearm storage patterns among dementia caregivers. To address this gap, this study examines firearm ownership and storage behaviors among dementia caregivers using data from the Behavioral Risk Factor Surveillance System (BRFSS). Specifically, we assess the prevalence of firearms in dementia caregivers’ households and identify demographic, health-related, and caregiving factors associated with having a loaded firearm and, among those with a loaded firearm, unlocked storage in the caregiver respondent’s household. Additionally, we explore interaction effects to determine how these factors jointly influence firearm storage practices.

## Methods

### Data and sample

This study used data from the BRFSS, a state-based, cross-sectional survey conducted annually by the Centers for Disease Control and Prevention (CDC) [22]. The BRFSS collects self-reported health-related behaviors, chronic conditions, and preventive health practices among U.S. adults through telephone-based surveys. Since both firearm storage and caregiving modules are optional and administered only in select states each year, this study combined four years of BRFSS data (2017, 2021, 2022, and 2023) to increase sample size and statistical power [23]. Only states that included both the firearm storage and caregiving modules were included in the analysis. The final state-year combinations were: 2017: Oregon; 2021: Alaska, New Mexico, North Carolina, Ohio; 2022: Ohio; 2023: Arizona, Idaho, Louisiana, Oregon. The data were accessed in August 2024 for research purposes. Authors did not have access to any identifiable private information or participant-level identifiers at any time. This study used publicly available, de-identified secondary data, which is exempt from human subjects review and does not require IRB approval.

The study sample included adults aged 18 years and older who reported household firearm ownership and answered the firearm storage and caregiving-related questions. Dementia caregivers were identified based on self-reported caregiving status, defined as providing regular care or assistance to a family member or friend within the past 30 days. Care recipients were classified as having ADRD if ADRD was reported as the main health problem or if ADRD was listed alongside another primary health condition. Non-caregivers were those who did not report providing such care. BRFSS does not indicate whether caregiver respondents co-resided with their care recipients. The final analytic sample consisted of 59,673 participants, of whom 3,436 were identified as dementia caregivers.

### Measures

#### Household Firearm Ownership and Storage Practices.

Firearm-related variables were assessed using BRFSS firearm ownership and storage questions. **Household Firearm Ownership**: Respondents were asked, “Are any firearms kept in or around your home?” (Yes/No). **Firearm Loaded**: Respondents who reported that a firearm was kept in or around their home were further asked, “Are any of these firearms currently loaded?” (Yes/No). **Firearm Locked**: Those who reported having a loaded firearm were asked, “Are all of these firearms kept in a locked place?” (Yes/No). Based on these responses, we compared the proportion of respondents reporting a firearm in the household between dementia caregivers and non-caregivers, estimated the proportion reporting a loaded firearm among dementia caregivers in firearm-owning households, and estimated the proportion reporting unlocked storage among dementia caregivers with a loaded firearm in the household [24].

#### Predicting Variables.

The selection of predictor variables was informed by prior research on firearm storage behaviors using BRFSS data, as well as the Stress Process Model (SPM) for caregiving-related factors.

**Demographic characteristics** including age (18–49, 50–64, 65+), sex (male/female), race/ethnicity (Non-Hispanic White, Black, Hispanic, Other), annual household income (≤$50K vs. > $50K), education (high school or below, some college, college or above), marital status (married/partnered vs. single), rural vs. urban residence, and veteran status (yes/no). These factors were included based on their established associations with household firearm ownership and storage practices [24–26]. Household income was dichotomized at $50,000 to create a consistent, parsimonious measure across the pooled BRFSS survey years while maintaining adequate cell sizes for subgroup and interaction analyses. The threshold corresponds to a boundary in the BRFSS income response categories and was not intended to represent a sample median.

**Health-related factors**, including self-rated health (good/better vs. fair/poor), number of chronic conditions, depression diagnosis (yes/no), and binge drinking behavior (yes/no), were included based on evidence linking physical and mental health conditions to firearm safety practice [26,27].

**Caregiving-related factors** were selected with reference to the Stress Process Model (SPM) to characterize caregiving context and care demands rather than to represent direct determinants of firearm-storage decisions [28–30]. In this study, we included caregiver’s relationship to care recipient (spouse, adult child, other relative, non-relative), caregiving duration (<2 years, 2–5 years, > 5 years), caregiving intensity (≤20 hours/week vs. > 20 hours/week), and assistance with personal care and/or household tasks.

### Analysis

This study combined four years of BRFSS data (2017, 2021, 2022, and 2023) from states that included both firearm storage and caregiving modules. To account for the complex survey design, computed sampling weights were applied, adjusting for state-level differences in representation across the four survey waves [31]. Additionally, year effects were controlled for in all regression models to account for potential temporal variations.

Descriptive analyses were conducted to compare household firearm ownership prevalence between dementia caregivers and non-caregivers using weighted chi-square tests. Among dementia caregivers, sample characteristics were summarized separately for those living in firearm-owning households and a subsample of those who had a loaded firearm in the home. Categorical variables were presented as frequencies and weighted percentages, while continuous variables were reported as means and standard deviations (SDs).

Two weighted logistic regression models were conducted. Model 1 examined the association between having a loaded firearm in the household and demographic, health-related, and caregiving factors among dementia caregivers. Model 2 examined the association between having a loaded and unlocked firearm among dementia caregivers using the same set of predictors. Interaction effects between demographics, health conditions, and caregiving characteristics were tested in both regression models. We excluded 56 (3.69%) participants from the analyses due to missing data on predictor variables, which was considered unlikely to meaningfully affect statistical power or bias estimates [32]. All analyses were conducted using STATA SE version 18 [33].

## Results

### Sample characteristics

This study analyzed 59,673 (weighted N = 7,566,023) respondents from BRFSS data (2017, 2021, 2022, and 2023), of whom 3,436 (weighted N = 444,744) were identified as dementia caregivers. Weighted analysis revealed that dementia caregivers had a significantly higher prevalence of household firearm ownership compared to non-caregivers (40.57% vs. 35.12%, χ^2^ = 27.47, p < 0.01).

Among dementia caregivers in households with firearms (n = 1,460), approximately 35.79% (n = 514) reported having a loaded firearm at home. Of this subgroup, 50.78% also reported that the loaded firearm was stored unlocked, representing the highest-risk storage condition. Table 1 presents the descriptive characteristics of dementia caregivers in firearm-owning households, and the subgroup with loaded firearms at home.

**Table 1:** Descriptive statistics of dementia caregivers in the analytic sample.

Variable		Firearm owning households (n = 1460)	Firearm loaded households (n = 514)
		N (weighted %) / [Mean (SD)]	N (weighted %) / [Mean (SD)]
Firearm loaded		514 (35.79%)	/
Firearm loaded and unlocked		/	270 (50.78%)
Age	18-49	341 (34.84%)	116 (38.24%)
	50-64	576 (38.69%)	206 (25.97%)
	65+	543 (26.46%)	192 (25.79%)
Sex (male)		583 (43.65%)	262 (53.22%)
Race/Ethnicity	Non-Hispanic White	1223 (79.61%)	426 (74.53%)
	Non-Hispanic Black	58 (9.74%)	26 (13.55%)
	Hispanic	75 (5.62%)	22 (6.21%)
	Other	104 (5.02%)	40 (5.71%)
Education	High school or below	371 (35.00%)	133 (36.46%)
	Some college	489 (36.51%)	179 (33.86%)
	College or above	598 (28.49%)	201 (29.68%)
Marital status (married/partnered)		1040 (69.66%)	341 (64.48%)
Residential area (rural)		181 (7.27%)	66 (7.19%)
Annual household income (>50K USD)		819 (62.77%)	285 (65.04%)
Veteran (yes)		193 (13.95%)	86 (15.42%)
Self-rated health (good/better)		1143 (77.45%)	390 (72.76%)
Binge drinker (yes)		187 (14.08%)	69 (17.12%)
Depression (yes)		429 (30.35%)	145 (28.56%)
No. of chronic conditions		[2.19 (1.66)]	[2.31 (1.72)]
Relationship type	Adult child	732 (56.86%)	252 (57.04%)
	Spouse	273 (15.43%)	87 (14.53%)
	Other relative	261 (17.64%)	98 (18.82%)
	Non-relative	194 (10.08%)	77 (9.62%)
Care duration	<2 years	578 (41.21%)	214 (40.52%)
	2-5 years	417 (28.85%)	138 (28.79%)
	>5 years	440 (29.94%)	153 (30.69%)
Care hours (>20 hours/week)		544 (40.45%)	177 (34.73%)
Helping with personal care (yes)		906 (65.51%)	302 (64.91%)
Helping with household tasks (yes)		1236 (87.93%)	423 (86.91%)

In the broader group of dementia caregivers with firearms in the home, most were Non-Hispanic White (79.61%), aged 50–64 years (38.69%), and married or partnered (69.66%). About 43.65% were male, and 62.77% reported an annual household income above $50K. Regarding health characteristics, 77.45% rated their health as good or better, 30.35% had a depression diagnosis, and the average number of chronic conditions was 2.19 (SD = 1.66). In terms of caregiving-related factors, 56.86% were adult children of the care recipient, while 15.43% were spousal caregivers. The duration of caregiving varied, with 41.21% providing care for less than two years, 28.85% for 2–5 years, and 29.94% for more than five years. Additionally, 40.45% of caregivers provided more than 20 hours of care per week, 65.51% helped with personal care and 87.93% helped with household tasks.

Compared to the overall group of dementia caregivers in firearm-owning households, those in households with a loaded firearm were more likely to be male (53.22%) and from higher-income households (>$50K: 65.04%). They also reported slightly lower levels of good or better self-rated health (72.76%). Caregiving characteristics were largely similar across the two groups, with one exception: a lower proportion of caregivers in households with a loaded firearm provided more than 20 hours of care per week (34.73%), compared to the overall group of dementia caregivers in firearm-owning households.

### Firearm loaded among dementia caregivers

A weighted logistic regression (unweighted N = 1460, weighted N = 180,415) analysis examined factors associated with having a loaded firearm in the household among dementia caregivers living in a household with a firearm (Table 2). The results indicate that male caregivers were significantly more likely to live in a household with a loaded firearm compared to female caregivers (OR=1.98, 95%CI: 1.32–2.96, p < 0.01). No other variables were significantly associated with keeping firearm loaded in the household.

**Table 2:** Factors associated with having a loaded firearm in the household among dementia caregivers (unweighted N = 1460, weighted N = 180,415).

		Odds Ratio	95%CI	p
Age	49 and younger	ref.	–	–
	50-64	0.91	(0.56, 1.50)	0.72
	65 and older	1.07	(0.60, 1.91)	0.83
Sex (male)		1.98	(1.32, 2.96)	0.001
Race/Ethnicity	Non-Hispanic White	ref.	–	–
	Non-Hispanic Black	1.32	(0.61, 2.85)	0.48
	Hispanic	0.62	(0.25, 1.54)	0.30
	Other	1.39	(0.66, 2.92)	0.39
Education Level	High school or below	ref.	–	–
	Some college	1.04	(0.63, 1.73)	0.87
	College or above	1.34	(0.80, 2.23)	0.26
Marital status (married/partnered)		0.62	(0.38, 1.01)	0.05
Residential area (rural)		1.36	(0.73, 2.53)	0.33
Annual household income (>50K USD)		1.55	(0.98, 2.43)	0.06
Veteran (yes)		0.60	(0.36, 1.02)	0.06
Self-rated health (good/better)		0.66	(0.37, 1.17)	0.15
Binge drinker (yes)		1.13	(0.65, 1.97)	0.66
Depression (yes)		0.83	(0.52, 1.34)	0.45
No. of chronic conditions		1.06	(0.92, 1.21)	0.41
Relationship Type	Adult child	ref.	–	–
	Spouse	0.82	(0.44, 1.54)	0.54
	Other relative	0.78	(0.46, 1.33)	0.36
	Non-relative	0.86	(0.48, 1.54)	0.60
Care duration	<2 years	ref.	–	–
	2-5 years	1.04	(0.63, 1.70)	0.88
	>5 years	1.28	(0.81, 2.00)	0.29
Care hours (>20 hours/week)		0.75	(0.48, 1.18)	0.21
Helping with personal care (yes)		0.88	(0.57, 1.36)	0.56
Helping with household tasks (yes)		0.70	(0.41, 1.19)	0.19

We then examined interaction effects between demographics, health conditions, and caregiving characteristics to assess how these factors jointly influence having a loaded firearm in the household. Three significant interactions were identified between (1) sex and caregiving duration (male * > 5 years caregiving, OR=2.57, 95%CI: 1.06–6.25, p = 0.04), (2) sex and caregiving intensity (male * > 20 hours care/week, OR=3.03, 95%CI: 1.34–6.83, p = 0.01), (3) sex and annual household income (male * > $50k annually, OR=2.59, 95%CI: 1.19–5.64, p = 0.02).

Specifically, among male caregivers, the probability of living in a household with loaded firearms increased with caregiving duration (53.21% for >5 years vs. 36.10% for <2 years), while for female caregivers, there is a slight decline in probability (27.44% for >5 years vs. 31.29% for <2 years). Male caregivers who provided more than 20 hours of care per week were more likely to live in a household with loaded firearms (49.21%) compared to those providing fewer hours (41.79%), while female caregivers providing more than 20 hours per week showed a decreased likelihood of living in a household with loaded firearms (20.53%) comparing to those providing fewer hours (35.39%). Male caregivers in higher-income households (>$50K) were more likely to live in a household with loaded firearms (50.55%) than lower-income male caregivers (29.55%). However, female caregivers’ likelihood of living in a household with loaded firearms remained consistent across income levels (28.83% in lower-income vs. 28.75% in higher-income households). Fig 1 presents predictive margins with 95% confidence intervals for the three significant interactions.

**Fig 1:**
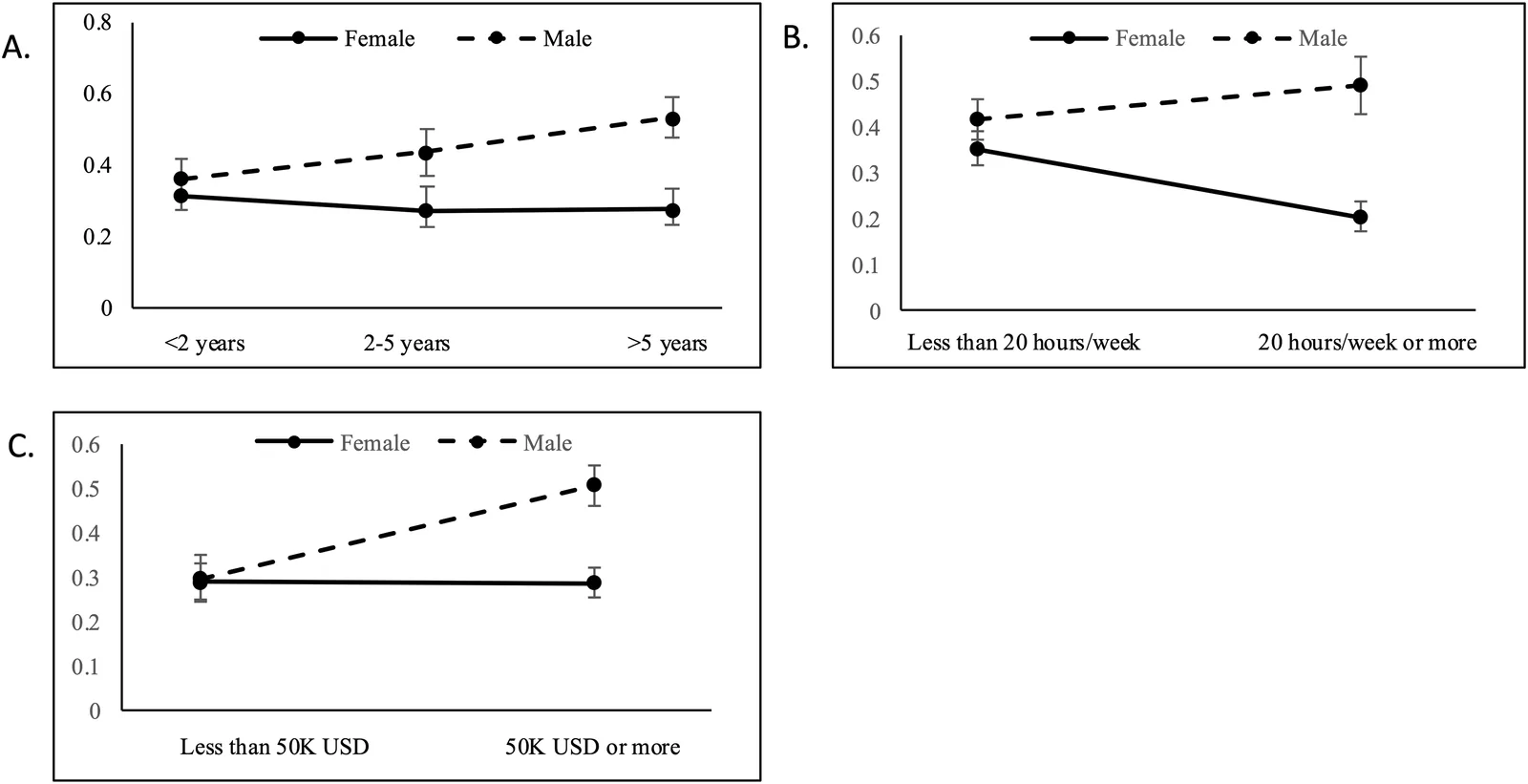
Predicted probabilities of having loaded firearms in households among dementia caregivers by key interactions. Panel A. Care duration * sex, Panel B. Care intensity * sex, Panel C. Annual household income * sex.

### Firearm unlocked among dementia caregivers

A weighted logistic regression (unweighted N = 514, weighted N = 64,181) examined factors associated with having unlocked firearms in the household among dementia caregivers who reported storing a firearm loaded (Table 3). The results indicate that older age was significantly associated with a higher risk of living in a household with loaded unlocked firearms. Compared to younger caregivers (18–49 years), caregivers aged 50–64 years had 3.74 times higher odds of living in a household with loaded unlocked firearms (OR=3.74, 95%CI: 1.75–8.01, p < 0.01), while caregivers aged 65 years and older had 5.39 times higher odds (OR=5.39, 95%CI: 2.02–14.40, p < 0.01). Longer caregiving duration and household task assistance were associated with lower odds of living in a household with loaded unlocked firearms. Caregivers who had provided care for more than five years had a significantly lower likelihood of living in a household with loaded unlocked firearms compared to those caregiving for less than two years (OR=0.36, 95%CI: 0.16–0.78, p = 0.01). Similarly, caregivers who reported helping with household tasks had a lower odds of living in a household with loaded unlocked firearms (OR=0.27, 95%CI: 0.11–0.63, p < 0.01).

**Table 3:** Factors associated with having loaded and unlocked firearms in the household among dementia caregivers (unweighted N = 514, weighted N = 64,181).

		Odds Ratio	95%CI	p
Age	49 and younger	ref.	–	–
	50-64	3.74	(1.75, 8.01)	0.001
	65 and older	5.39	(2.02, 14.40)	0.001
Sex (male)		0.80	(0.41, 1.55)	0.50
Race/Ethnicity	Non-Hispanic White	ref.	–	–
	Non-Hispanic Black	2.09	(0.67, 6.51)	0.20
	Hispanic	1.39	(0.40, 4.86)	0.61
	Other	2.22	(0.67, 7.35)	0.19
Education Level	High school or below	ref.	–	–
	Some college	1.34	(0.62, 2.89)	0.46
	College or above	1.20	(0.52, 2.77)	0.67
Marital status (married/partnered)		0.56	(0.28, 1.10)	0.09
Residential area (rural)		1.95	(0.64, 5.95)	0.24
Annual household income (>50K USD)		1.01	(0.46, 2.20)	0.99
Veteran (yes)		0.99	(0.38, 2.57)	0.98
Self-rated health (good/better)		1.46	(0.65, 3.27)	0.36
Binge drinker (yes)		1.42	(0.57, 3.49)	0.45
Depression (yes)		1.76	(0.81, 3.79)	0.15
No. of chronic conditions		0.90	(0.73, 1.11)	0.32
Relationship Type	Adult child	ref.	–	–
	Spouse	2.10	(0.84, 5.30)	0.11
	Other relative	0.80	(0.39, 1.64)	0.54
	Non-relative	1.03	(0.33, 3.24)	0.95
Care duration	<2 years	ref.	–	–
	2-5 years	0.58	(0.25, 1.35)	0.21
	>5 years	0.36	(0.16, 0.78)	0.01
Care hours (>20 hours/week)		0.53	(0.26, 1.08)	0.08
Helping with personal care (yes)		0.69	(0.33, 1.43)	0.31
Helping with household tasks (yes)		0.27	(0.11, 0.63)	0.003

We then examined interaction effects between demographic, health, and caregiving factors to assess how these variables jointly influence firearm unlocking behaviors. Three significant interactions were identified: (1) age and length of caregiving (50–64 years * 2–5 years caregiving, OR=8.78, 95%CI: 1.44–35.72, p = 0.02), (2) age and helping with household tasks (50–64 years * providing household assistance, OR=3.15, 95%CI: 1.28–8.60, p < 0.01; 65 + * providing household assistance, OR=1.90, 95%CI: 1.30–3.43, p = 0.03), and (3) sex and annual household income (male * > $50K annually, OR=4.50, 95%CI: 1.16–7.15, p = 0.03).

Specifically, younger caregivers (<50 years) with more caregiving experience were less likely to live in a household with unlocked firearms (15.75% for >5 years vs. 50.52% for <2 years), whereas older caregivers (50–64 and 65+) showed higher probabilities of living in a household with unlocked firearms, even with increased caregiving experience (49.14% for >5 years in the 50–64 group; 48.94% in the 65 + group). Caregivers under 50 who assisted with household tasks were significantly less likely to live in a household with unlocked firearms (21.28%), while the probability remained stable for older caregivers (53.98% for 50–64 years and 58.07% for 65 + years). Additionally, male caregivers in higher-income households (>$50K) had an increased probability of living in a household with unlocked firearms (47.83%) compared to their lower-income counterparts (33.51%), whereas female caregivers demonstrated a decreasing trend (56.87% in lower-income vs. 43.94% in higher-income households). Fig 2 presents predictive margins with 95% confidence intervals for these three significant interactions.

**Fig 2:**
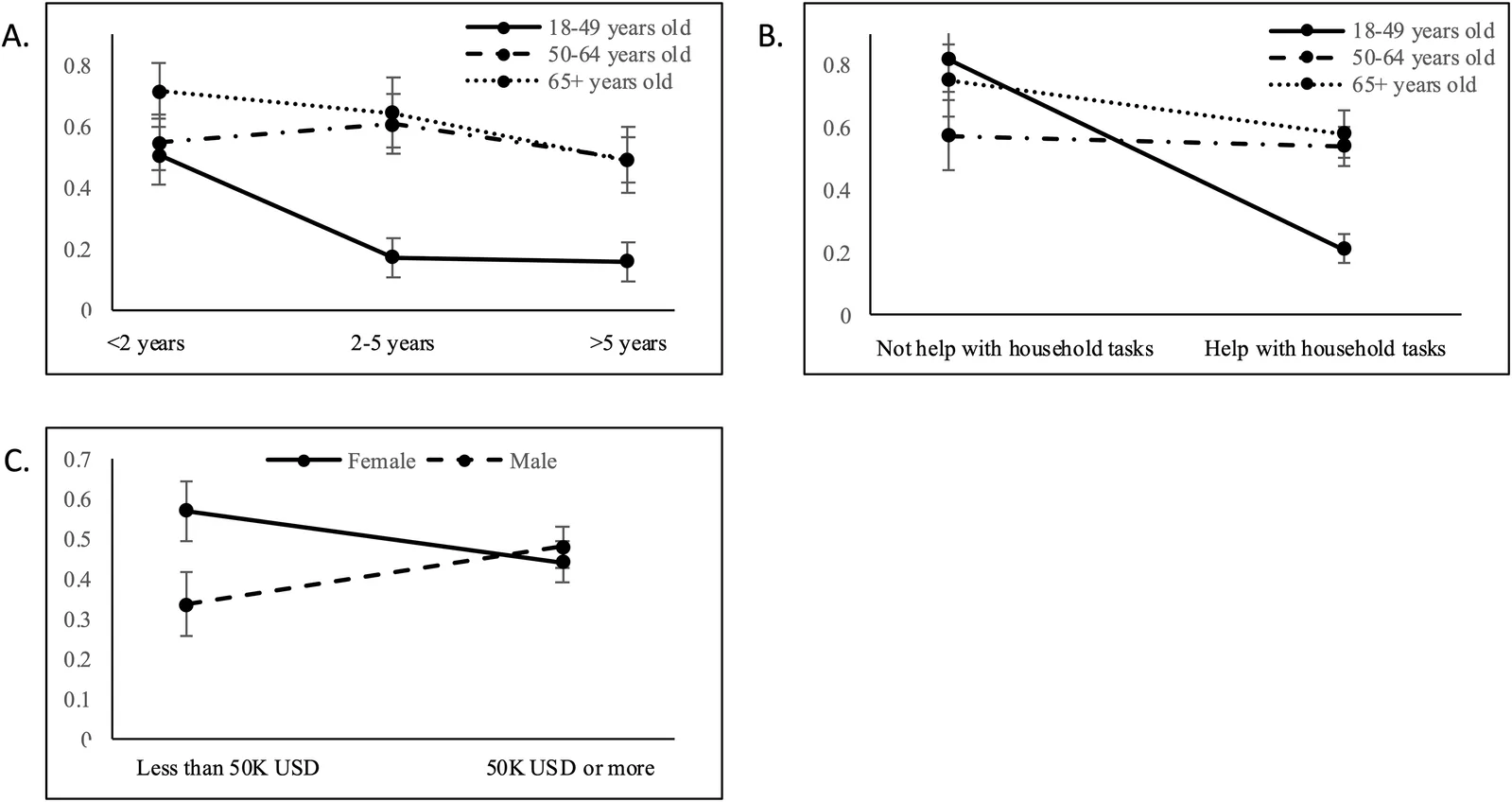
Predicted probabilities of having unlocked firearms in households among dementia caregivers by key interactions. Panel A. Care duration * age, Panel B. Helping with household tasks* age, Panel C. Annual household income * sex.

## Discussion

This study pooled four years of BRFSS data, offering one of the first large-scale examinations of firearm storage practices among dementia caregivers. Findings indicate that household firearm ownership is significantly higher among dementia caregivers than non-caregivers, with many reporting that having firearms loaded (35.8%) and unlocked (50.8%) at home, potentially increasing the risk of firearm-related injury or violence for individuals with dementia and their families. However, because caregiver–care recipient co-residence and care-recipient firearm access were not measured, these estimates should not be interpreted as direct measures of firearm exposure among people living with dementia.

Consistent with previous research, this study also found that older age and being male were associated with lower adherence to secure firearm storage practices [21,26]. Specifically, older dementia caregivers (50+) and male caregivers were significantly more likely to report having firearms both loaded and unlocked in their households. This may be linked to age-related declines in safety awareness as well as longstanding household firearm storage habits and self-defense motivations [34]. Additionally, an exploratory interaction between sex and household income indicated a higher predicted probability of unsafe storage among higher-income male caregivers. This finding differs from prior research suggesting that material hardship may act as a barrier to secure firearm storage [25]. This discrepancy may be related to differences in how financial hardship is measured, which includes difficulties across multiple domains, such as employment, food security, housing, and transportation. Despite related to household income, they are not equivalent. In this study, there is no direct association between income and household firearm storage. The relationship was found specifically through an interaction between income and sex. This may be explained that higher-income male caregivers may have greater long-term access to firearms, lower perceived risk, and reduced responsiveness to firearm safety messaging. However, because BRFSS only assesses household firearm ownership and not individual ownership, more detailed data are needed to clarify how financial conditions and specific material hardships influence individual firearm storage behaviors.

Prior research suggests that dementia caregivers may have greater awareness of firearm safety issues and a higher interest in receiving safety counseling from healthcare providers [15,35]. Our findings similarly indicate that longer caregiving duration and assisting with household chores were associated with safer household firearm storage practices. In interaction analyses, these associations with safer storage were primarily observed among younger caregivers, who were less likely to live in households with firearms unlocked when assisting with household tasks, whereas older caregivers’ household firearm storage practices remained unchanged. These findings suggest that firearm safety interventions should consider age-specific adaptations, ensuring that older caregivers receive targeted strategies to improve secure firearm storage habits.

### Public health implications

Given the prevalence of loaded and unlocked firearm storage among dementia caregivers, healthcare providers may consider firearm safety as part of broader dementia safety discussions. Such conversations should establish whether firearms are present in the caregiver’s or care recipient’s residence, whether the person living with dementia can access them, and who controls firearm storage [36]. Firearm safety programs may particularly consider age- and sex-related differences in storage practices while tailoring counseling to firearm presence, access, and household circumstances [15,35]. State and federal policies should promote safe firearm storage laws and tax incentives for firearm safety devices in caregiving households [37]. Future studies should directly assess firearm attitudes, reasons for firearm ownership, perceived risk, caregiver stress, and decision-making to clarify mechanisms underlying firearm-storage practices among dementia caregivers. Integrating firearm safety education into caregiver support groups, alongside existing stress-reduction content, may provide an effective intervention strategy.

### Limitations

This study has several limitations. Its cross-sectional design prevents causal inference between caregiving and firearm storage behaviors. Second, data were only available from select states, as the firearm and caregiving modules were optional in BRFSS, limiting generalizability to all dementia caregivers in the U.S. Importantly, BRFSS does not indicate whether caregiver respondents co-resided with their care recipients. Therefore, a firearm reported in the caregiver’s household cannot be assumed to have been present in the care recipient’s residence or accessible to the person living with dementia. Lastly, the survey lacked detailed information on firearm storage and access, such as who owns firearms, how firearms were stored, the extent to which older adults with dementia had access, and the severity of the older adult’s cognitive impairment. These factors are critical for assessing firearm-related risks and understanding mechanisms for promoting safe storage within caregiving dyads. Future research should integrate more detailed caregiver-recipient dyadic assessments to explore firearm accessibility, cognitive status, and decision-making processes related to firearm safety interventions.

## Conclusion

This study highlights the high prevalence of household firearm ownership among dementia caregivers and identifies key risk and protective factors associated with unsafe storage practices. Findings support continued attention to firearm safety in dementia caregiving while underscoring the importance of considering household circumstances, living arrangements, and firearm access when developing counseling and prevention strategies. Given the risks posed by firearm access in dementia care settings, policy efforts, healthcare interventions, and caregiver education are essential to promoting safe storage practices and reducing firearm-related harm in caregiving households.
