# Supplementary material for: Unsafe firearm storage among family caregivers of older persons living with dementia
Source: PLoS One. 2026 Oct 28;21(10):e0360412. doi: 10.1371/journal.pone.0360412 (PMC13716227; doi:10.1371/journal.pone.0360412)
Supplement: Supplementary file 1 [file pone.0360412.s002.docx]

Dear Dr. Akintomide and Reviewers,

Thank you for the opportunity to revise our manuscript, “*Unsafe Firearm Storage Among Family Caregivers of Older Persons Living With Dementia*” (PONE-D-25-62908). We appreciate the careful and constructive comments from both reviewers.

We have revised the manuscript to address all comments. The principal revisions include clarifying that the BRFSS firearm measures characterize the caregiver respondent’s household and do not establish co-residence with or firearm access by the care recipient; refining the role of the Stress Process Model in selecting caregiving-related variables; clarifying the rationale for the household income categorization; correcting and refining the Abstract and references; adding keywords; revising the public health implications to avoid overinterpreting the income interaction; and updating the Data Availability Statement with direct links to each BRFSS dataset.

A detailed point-by-point response is provided below. All changes are shown in the tracked-changes manuscript, and a clean revised manuscript has also been provided.

**ACADEMIC EDITOR**

Journal Requirement 1. PLOS ONE formatting requirements

**Response:** Thank you. We reviewed the manuscript against the PLOS ONE formatting requirements and revised the manuscript accordingly.

Journal Requirement 2. Data Availability Statement

**Response:** Thank you. We have updated the Data Availability Statement to provide direct links to the CDC BRFSS data. These datasets are publicly available and require no special permission for access.

**REVIEWER #1**

Comment 1. Co-residence of caregivers and care recipients

**Response:** Thank you for identifying this important limitation. We agree that the BRFSS data do not indicate whether the caregiver respondent co-resided with the person living with dementia. The firearm questions refer specifically to firearms kept in or around the respondent’s home. Therefore, for caregivers who did not live with their care recipient, the reported firearm environment would characterize the caregiver’s household rather than the care recipient’s household, and the data cannot establish whether the person living with dementia had access to the reported firearm.

We have revised the manuscript throughout to make this distinction explicit. Specifically, we clarified in the Methods that the firearm measures characterize the caregiver respondent’s household and that BRFSS does not provide information on caregiver–care recipient co-residence. We also revised the Introduction and Discussion to avoid implying that the reported firearms were necessarily present in the care recipient’s residence or accessible to the person living with dementia. Finally, we added lack of information on co-residence as a prominent study limitation and emphasized the need for future dyadic research that directly assesses living arrangements, firearm ownership and control, and firearm access by the person living with dementia. These revisions are provided on [page 3, lines 67–71; page 4, lines 94–94; page 16, lines 303-305; page 17, lines 335-339; page 18, lines 359-362]. Here are the revised/added text:

*Introduction:*

*Dementia caregivers often provide assistance with medical needs, household tasks, personal care, and safety.[14] When firearms are present, caregivers may also encounter decisions related to firearm storage and access, although their role in such decisions depends on living arrangements, firearm ownership, and access.[15]*

*Specifically, we assess the prevalence of firearms in dementia caregivers’ households and identify demographic, health-related, and caregiving factors associated with having a loaded firearm and, among those with a loaded firearm, unlocked storage in the caregiver respondent’s household.*

*Discussion:*

*However, because caregiver–care recipient co-residence and care-recipient firearm access were not measured, these estimates should not be interpreted as direct measures of firearm exposure among people living with dementia.*

*Given the prevalence of loaded and unlocked firearm storage among dementia caregivers, healthcare providers may consider firearm safety as part of broader dementia safety discussions. Such conversations should establish whether firearms are present in the caregiver’s or care recipient’s residence, whether the person living with dementia can access them, and who controls firearm storage.[35]*

*Importantly, BRFSS does not indicate whether caregiver respondents co-resided with their care recipients. Therefore, a firearm reported in the caregiver’s household cannot be assumed to have been present in the care recipient’s residence or accessible to the person living with dementia.*

Comment 2. Use of the Stress Process Model

**Response:** Thank you for this observation. We agree that our original wording overstated the role of the Stress Process Model by implying that caregiving stress directly influences firearm-storage decision-making. Our intent was to use the Stress Process Model to inform the selection and organization of caregiving-context variables, including caregiver relationship, caregiving duration and intensity, and assistance with personal care and household tasks, rather than to test a causal pathway from caregiving stress to firearm storage.

We have revised the Methods accordingly and added an appropriate foundational reference for the Stress Process Model. We also removed language implying that caregiving stress directly leads to unsafe firearm use or storage. In addition, we now acknowledge that firearm attitudes, motivations for firearm ownership, perceived risk, and related factors may be more proximal determinants of firearm-storage decisions but are not measured in BRFSS. These revisions are provided on [page 7, lines 158–160; page 17-18, lines 342-353].

*Methods:*

*Caregiving-related factors were selected with reference to the Stress Process Model (SPM) to characterize caregiving context and care demands rather than to represent direct determinants of firearm-storage decisions.[28-30]*

*Discussion:*

*Future studies should directly assess firearm attitudes, reasons for firearm ownership, perceived risk, caregiver stress, and decision-making to clarify mechanisms underlying firearm-storage practices among dementia caregivers.*

**REVIEWER #2**

Comment 1. Abstract wording

**Response:** Thank you. We corrected the grammatical errors identified in the Abstract. We also reviewed the Abstract for consistency with the reported analyses and clarified the description of caregiving duration and the study conclusions. The revised abstract is here:

*Abstract*

*Purpose: Firearm access in dementia caregiving households poses safety risks. This study examines household firearm ownership and storage practices among dementia caregivers, identifying factors associated with unsafe firearm storage.*

*Methods: We analyzed pooled data from 2017, 2021, 2022, and 2023 Behavioral Risk Factor Surveillance System (BRFSS). Weighted logistic regression assessed associations between caregiver characteristics and firearm storage behaviors (unloaded vs. loaded, locked vs. unlocked).*

*Results: Dementia caregivers were more likely to live in firearm-owning households than non-caregivers (40.57% vs. 35.12%). Among caregivers in firearm-owning households, 35.8% reported loaded storage, and 50.8% of those also reported unlocked storage. Older caregivers are more likely to report unlocked firearm storage (50–64 years: OR=3.74, 95%CI: 1.75–8.01; 65+ years: OR=5.39, 95%CI: 2.02–14.40; reference: <50 years). Male caregivers had higher odds of reporting a loaded firearm in the household (OR=1.98, 95% CI: 1.32–2.96). In contrast, longer caregiving duration (>5 years: OR=0.36, 95%CI: 0.16–0.78) and assisting with household tasks (OR=0.27, 95%CI: 0.11–0.63) were associated with safer storage, though this protective effect was only observed among younger caregivers.*

*Conclusion: Household firearm storage patterns among dementia caregivers varied by caregiver age, sex, and caregiving context, highlighting the need for tailored firearm-safety counseling and caregiver-focused public health strategies.*

Comment 2. Keywords

**Response:** Thank you. We have added keywords to the manuscript.

*Keywords: dementia; caregiving; firearm storage; injury prevention; Behavioral Risk Factor Surveillance System*

Comment 3. Reference 1 and the estimated number of firearms

**Response:** Thank you for identifying this issue. We reviewed the original Small Arms Survey source. The approximately 857 million estimate refers to civilian-held firearms globally, whereas the corresponding 2017 estimate for the United States was approximately 393 million civilian-held firearms, or approximately 120.5 firearms per 100 residents. We have updated Reference 1 to link directly to the primary Small Arms Survey report PDF.

Comment 4. “Proportion of household”

**Response:** Thank you. We corrected “proportion household” to “proportion of households.” We also revised the surrounding firearm-measure language to consistently indicate that the BRFSS questions assess firearms in the respondent’s household rather than individual firearm ownership.

Comment 5. Rationale for the $50,000 household income cutoff

**Response:** Thank you for requesting clarification. The $50,000 threshold was not intended to represent the median income cutoff used in Reference 25. Rather, income was dichotomized to provide a consistent and parsimonious measure across the pooled BRFSS survey years and to maintain adequate cell sizes for subgroup and interaction analyses. The $50,000 threshold corresponds to a boundary in the BRFSS income response categories used to construct the harmonized measure. We have added this rationale to the Methods and clarified that Reference 25 supported inclusion of household economic characteristics generally rather than selection of this specific cutoff [Page 6, lines 143-147]. Revised text is here:

*Methods:*

*Household income was dichotomized at $50,000 to create a consistent, parsimonious measure across the pooled BRFSS survey years while maintaining adequate cell sizes for subgroup and interaction analyses. The threshold corresponds to a boundary in the BRFSS income response categories and was not intended to represent a sample median.*

Comment 6. Practical targeting of higher-income caregivers

**Response:** Thank you for raising this point. Upon reviewing the interpretation, we agree that the findings do not support recommending interventions targeted broadly to higher-income caregivers. Household income was not independently associated with unlocked firearm storage; rather, income appeared in an exploratory interaction with sex. We have therefore removed higher-income caregivers from the groups recommended for targeted intervention and revised the Abstract, Discussion, and Public Health Implications accordingly. The sex-by-income interaction remains reported as an exploratory finding, but we now emphasize that it requires further investigation rather than using it as a basis for income-targeted intervention [Page 17, lines 339-341; Page 18, lines 372-373]. Revised text is here:

*Discussion:*

*Firearm safety programs may particularly consider age- and sex-related differences in storage practices while tailoring counseling to firearm presence, access, and household circumstances.[15,34]*

*Findings support continued attention to firearm safety in dementia caregiving while underscoring the importance of considering household circumstances, living arrangements, and firearm access when developing counseling and prevention strategies.*
