# Supplementary material for: Unsafe firearm storage among family caregivers of older persons living with dementia
Source: PLoS One. 2026 Oct 28;21(10):e0360412. doi: 10.1371/journal.pone.0360412 (PMC13716227; doi:10.1371/journal.pone.0360412)
Supplement: Peer review history

# Peer review: Decision Letter 0

**DOI:** 10.1371/journal.pone.0360412.r001

**Authors:** Modupe Akintomide

13 Jul 2026

Dear Dr. Liang,

plosone@plos.org. When you're ready to submit your revision, log on to https://www.editorialmanager.com/pone/ and select the 'Submissions Needing Revision' folder to locate your manuscript file.

- A letter that responds to each point raised by the academic editor and reviewer(s). You should upload this letter as a separate file labeled 'Response to Reviewers'.
- A marked-up copy of your manuscript that highlights changes made to the original version. You should upload this as a separate file labeled 'Revised Manuscript with Track Changes'.
- An unmarked version of your revised paper without tracked changes. You should upload this as a separate file labeled 'Manuscript'.

If you would like to make changes to your financial disclosure, please include your updated statement in your cover letter. Guidelines for resubmitting your figure files are available below the reviewer comments at the end of this letter.

As the corresponding author, your ORCID iD is verified in the submission system and will appear in the published article. PLOS supports the use of ORCID, and we encourage all coauthors to register for an ORCID iD and use it as well. Please encourage your coauthors to verify their ORCID iD within the submission system before final acceptance, as unverified ORCID iDs will not appear in the published article. *Only*  the individual author can complete the verification step; PLOS staff *cannot*  verify ORCID iDs on behalf of authors.

We look forward to receiving your revised manuscript.

Kind regards,

Modupe Akintomide

Academic Editor

PLOS One

Journal Requirements:

When submitting your revision, we need you to address these additional requirements.

1. Please ensure that your manuscript meets PLOS ONE's style requirements, including those for file naming. The PLOS ONE style templates can be found at

https://journals.plos.org/plosone/s/file?id=wjVg/PLOSOne_formatting_sample_main_body.pdf and

https://journals.plos.org/plosone/s/file?id=ba62/PLOSOne_formatting_sample_title_authors_affiliations.pdf

2. Please note that your Data Availability Statement is currently missing the DOI/accession number of each dataset or a direct link to access each database. If your manuscript is accepted for publication, you will be asked to provide these details on a very short timeline. We therefore suggest that you provide this information now, though we will not hold up the peer review process if you are unable.

3. If the reviewer comments include a recommendation to cite specific previously published works, please review and evaluate these publications to determine whether they are relevant and should be cited. There is no requirement to cite these works unless the editor has indicated otherwise.

Reviewers' comments:

Reviewer's Responses to Questions

**Comments to the Author**

1. Is the manuscript technically sound, and do the data support the conclusions?

Reviewer #1: No

Reviewer #2: Yes

**********

2. Has the statistical analysis been performed appropriately and rigorously?

Reviewer #1: No

Reviewer #2: Yes

**********

3. Have the authors made all data underlying the findings in their manuscript fully available?

The PLOS Data policy

Reviewer #1: Yes

Reviewer #2: Yes

**********

4. Is the manuscript presented in an intelligible fashion and written in standard English?

Reviewer #1: Yes

Reviewer #2: Yes

**********

Reviewer #1: In this study, the authors analyzed the multi-year and multi-state BRFSS data to examine the dementia caregiver characteristics that were associated with unsafe storage of firearms. The study is well-intended, but it has a major problem.

1. The authors listed the BRFSS data constraints, such as who owns firearms, how firearms were stored, the extent to which older adults with dementia had access, and the severity of the older adult’s cognitive impairment. However, the biggest limitation that the authors did not list is if the caregiver was co-residing with the care recipient. The study showed that 57% of the caregivers (BRFSS respondents) were children of the older adults with dementia. How many of them were living with their parent-care recipients (i.e., in the same households)? For those who did not co-reside with the care recipients, the firearm-related data were applicable to their own households, not the care recipients’ households. How would you explain why the firearms in their own households may pose any danger for/by the care recipients? A study of older firearm suicide decedents found that some family members who did not live with the older adults did not even know that the older adults had kept any firearm. Other family members knew but they could not find where the older adults kept the firearms.

2. The Stress Processing Model (SPM): The authors need to provide more justifications for applying this theory. How does caregiving stress affect their firearm-related decision-making? I do not know if that is likely. More likely factors are attitudes toward firearms.

Reviewer #2: Thank you for the opportunity to review your article. This is an interesting study that adds to the evidence base around firearm ownership and (un)safe storage among carers of people living with dementia.

Here are my observations and feedback.

ABSTRACT: In the conclusion section, "unsafely firearm storage" should read "unsafe firearm storage" and "highlight" should be "highlighting"

KEYWORDS need to be added.

REFERENCES: The link to reference #1 currently does not lead to the main document - needs updating. The reference material found elsewhere on search reports approximately 857 million civilian-held firearms in the world at the end of 2017, which varies from what has been reported in the introduction - please check.

METHODS:

- Line 108: Should be proportion "of" household..

- Line 117-118: Why was the income cut-off in particular set at $50K? The current justification is "These factors were included based on their established associations with household firearm ownership and storage practices.[24–26]". However, looking at the figures presented in reference material 25 (that uses income variable), $50K wasn't the median income cut-off in their study. It would therefore be helpful to explain why the cut-off was set at $50K for this study.

- Line 274-286: The justification provided for the significant interaction finding is satisfactory. The measurement metrices are different from previous study as income is assessed as a standalone variable and the firearm ownership is a household level variable (not caregiver specific - even though there can be justification that the household member with a non-dementia diagnosis is likely to be the licenced owner too, but the dataset does not have that level of detail available, which has also been mentioned as a limitation).

- Line 293-295: Important public health messaging recommendation.

- Line 299: Are there specific practical ways that healthcare providers can particularly target higher-income carers for messaging? Practical suggestions on the how would be helpful.

**********

what does this mean?). If published, this will include your full peer review and any attached files.

If you choose “no”, your identity will remain anonymous but your review may still be made public.

**Do you want your identity to be public for this peer review?**  For information about this choice, including consent withdrawal, please see our Privacy Policy

Reviewer #1: No

Reviewer #2: **Yes:**  Rupesh Gautam

**********

[NOTE: If reviewer comments were submitted as an attachment file, they will be attached to this email and accessible via the submission site. Please log into your account, locate the manuscript record, and check for the action link "View Attachments". If this link does not appear, there are no attachment files.]

To ensure your figures meet our technical requirements, please review our figure guidelines: https://journals.plos.org/plosone/s/figures

You may also use PLOS’s free figure tool, NAAS, to help you prepare publication quality figures: https://journals.plos.org/plosone/s/figures#loc-tools-for-figure-preparation.

NAAS will assess whether your figures meet our technical requirements by comparing each figure against our figure specifications.

# Author comment: Author response to Decision Letter 1

**DOI:** 10.1371/journal.pone.0360412.r002

10 Aug 2026

Dear Dr. Akintomide and Reviewers,

Thank you for the opportunity to revise our manuscript, “Unsafe Firearm Storage Among Family Caregivers of Older Persons Living With Dementia” (PONE-D-25-62908). We appreciate the careful and constructive comments from both reviewers.

We have revised the manuscript to address all comments. The principal revisions include clarifying that the BRFSS firearm measures characterize the caregiver respondent’s household and do not establish co-residence with or firearm access by the care recipient; refining the role of the Stress Process Model in selecting caregiving-related variables; clarifying the rationale for the household income categorization; correcting and refining the Abstract and references; adding keywords; revising the public health implications to avoid overinterpreting the income interaction; and updating the Data Availability Statement with direct links to each BRFSS dataset.

A detailed point-by-point response is provided below. All changes are shown in the tracked-changes manuscript, and a clean revised manuscript has also been provided.

ACADEMIC EDITOR

Journal Requirement 1. PLOS ONE formatting requirements

Response: Thank you. We reviewed the manuscript against the PLOS ONE formatting requirements and revised the manuscript accordingly.

Journal Requirement 2. Data Availability Statement

Response: Thank you. We have updated the Data Availability Statement to provide direct links to the CDC BRFSS data. These datasets are publicly available and require no special permission for access.

REVIEWER #1

Comment 1. Co-residence of caregivers and care recipients

Response: Thank you for identifying this important limitation. We agree that the BRFSS data do not indicate whether the caregiver respondent co-resided with the person living with dementia. The firearm questions refer specifically to firearms kept in or around the respondent’s home. Therefore, for caregivers who did not live with their care recipient, the reported firearm environment would characterize the caregiver’s household rather than the care recipient’s household, and the data cannot establish whether the person living with dementia had access to the reported firearm.

We have revised the manuscript throughout to make this distinction explicit. Specifically, we clarified in the Methods that the firearm measures characterize the caregiver respondent’s household and that BRFSS does not provide information on caregiver–care recipient co-residence. We also revised the Introduction and Discussion to avoid implying that the reported firearms were necessarily present in the care recipient’s residence or accessible to the person living with dementia. Finally, we added lack of information on co-residence as a prominent study limitation and emphasized the need for future dyadic research that directly assesses living arrangements, firearm ownership and control, and firearm access by the person living with dementia. These revisions are provided on [page 3, lines 67–71; page 4, lines 94–94; page 16, lines 303-305; page 17, lines 335-339; page 18, lines 359-362]. Here are the revised/added text:

Introduction:

Dementia caregivers often provide assistance with medical needs, household tasks, personal care, and safety.[14] When firearms are present, caregivers may also encounter decisions related to firearm storage and access, although their role in such decisions depends on living arrangements, firearm ownership, and access.[15]

Specifically, we assess the prevalence of firearms in dementia caregivers’ households and identify demographic, health-related, and caregiving factors associated with having a loaded firearm and, among those with a loaded firearm, unlocked storage in the caregiver respondent’s household.

Discussion:

However, because caregiver–care recipient co-residence and care-recipient firearm access were not measured, these estimates should not be interpreted as direct measures of firearm exposure among people living with dementia.

Given the prevalence of loaded and unlocked firearm storage among dementia caregivers, healthcare providers may consider firearm safety as part of broader dementia safety discussions. Such conversations should establish whether firearms are present in the caregiver’s or care recipient’s residence, whether the person living with dementia can access them, and who controls firearm storage.[35]

Importantly, BRFSS does not indicate whether caregiver respondents co-resided with their care recipients. Therefore, a firearm reported in the caregiver’s household cannot be assumed to have been present in the care recipient’s residence or accessible to the person living with dementia.

Comment 2. Use of the Stress Process Model

Response: Thank you for this observation. We agree that our original wording overstated the role of the Stress Process Model by implying that caregiving stress directly influences firearm-storage decision-making. Our intent was to use the Stress Process Model to inform the selection and organization of caregiving-context variables, including caregiver relationship, caregiving duration and intensity, and assistance with personal care and household tasks, rather than to test a causal pathway from caregiving stress to firearm storage.

We have revised the Methods accordingly and added an appropriate foundational reference for the Stress Process Model. We also removed language implying that caregiving stress directly leads to unsafe firearm use or storage. In addition, we now acknowledge that firearm attitudes, motivations for firearm ownership, perceived risk, and related factors may be more proximal determinants of firearm-storage decisions but are not measured in BRFSS. These revisions are provided on [page 7, lines 158–160; page 17-18, lines 342-353].

Methods:

Caregiving-related factors were selected with reference to the Stress Process Model (SPM) to characterize caregiving context and care demands rather than to represent direct determinants of firearm-storage decisions.[28-30]

Discussion:

Future studies should directly assess firearm attitudes, reasons for firearm ownership, perceived risk, caregiver stress, and decision-making to clarify mechanisms underlying firearm-storage practices among dementia caregivers.

REVIEWER #2

Comment 1. Abstract wording

Response: Thank you. We corrected the grammatical errors identified in the Abstract. We also reviewed the Abstract for consistency with the reported analyses and clarified the description of caregiving duration and the study conclusions. The revised abstract is here:

Abstract

Purpose: Firearm access in dementia caregiving households poses safety risks. This study examines household firearm ownership and storage practices among dementia caregivers, identifying factors associated with unsafe firearm storage.

Methods: We analyzed pooled data from 2017, 2021, 2022, and 2023 Behavioral Risk Factor Surveillance System (BRFSS). Weighted logistic regression assessed associations between caregiver characteristics and firearm storage behaviors (unloaded vs. loaded, locked vs. unlocked).

Results: Dementia caregivers were more likely to live in firearm-owning households than non-caregivers (40.57% vs. 35.12%). Among caregivers in firearm-owning households, 35.8% reported loaded storage, and 50.8% of those also reported unlocked storage. Older caregivers are more likely to report unlocked firearm storage (50–64 years: OR=3.74, 95%CI: 1.75–8.01; 65+ years: OR=5.39, 95%CI: 2.02–14.40; reference: <50 years). Male caregivers had higher odds of reporting a loaded firearm in the household (OR=1.98, 95% CI: 1.32–2.96). In contrast, longer caregiving duration (>5 years: OR=0.36, 95%CI: 0.16–0.78) and assisting with household tasks (OR=0.27, 95%CI: 0.11–0.63) were associated with safer storage, though this protective effect was only observed among younger caregivers.

Conclusion: Household firearm storage patterns among dementia caregivers varied by caregiver age, sex, and caregiving context, highlighting the need for tailored firearm-safety counseling and caregiver-focused public health strategies.

Comment 2. Keywords

Response: Thank you. We have added keywords to the manuscript.

Keywords: dementia; caregiving; firearm storage; injury prevention; Behavioral Risk Factor Surveillance System

Comment 3. Reference 1 and the estimated number of firearms

Response: Thank you for identifying this issue. We reviewed the original Small Arms Survey source. The approximately 857 million estimate refers to civilian-held firearms globally, whereas the corresponding 2017 estimate for the United States was approximately 393 million civilian-held firearms, or approximately 120.5 firearms per 100 residents. We have updated Reference 1 to link directly to the primary Small Arms Survey report PDF.

Comment 4. “Proportion of household”

Response: Thank you. We corrected “proportion household” to “proportion of households.” We also revised the surrounding firearm-measure language to consistently indicate that the BRFSS questions assess firearms in the respondent’s household rather than individual firearm ownership.

Comment 5. Rationale for the $50,000 household income cutoff

Response: Thank you for requesting clarification. The $50,000 threshold was not intended to represent the median income cutoff used in Reference 25. Rather, income was dichotomized to provide a consistent and parsimonious measure across the pooled BRFSS survey years and to maintain adequate cell sizes for subgroup and interaction analyses. The $50,000 threshold corresponds to a boundary in the BRFSS income response categories used to construct the harmonized measure. We have added this rationale to the Methods and clarified that Reference 25 supported inclusion of household economic characteristics generally rather than selection of this specific cutoff [Page 6, lines 143-147]. Revised text is here:

Methods:

Household income was dichotomized at $50,000 to create a consistent, parsimonious measure across the pooled BRFSS survey years while maintaining adequate cell sizes for subgroup and interaction analyses. The threshold corresponds to a boundary in the BRFSS income response categories and was not intended to represent a sample median.

Comment 6. Practical targeting of higher-income caregivers

Response: Thank you for raising this point. Upon reviewing the interpretation, we agree that the findings do not support recommending interventions targeted broadly to higher-income caregivers. Household income was not independently associated with unlocked firearm storage; rather, income appeared in an exploratory interaction with sex. We have therefore removed higher-income caregivers from the groups recommended for targeted intervention and revised the Abstract, Discussion, and Public Health Implications accordingly. The sex-by-income interaction remains reported as an exploratory finding, but we now emphasize that it requires further investigation rather than using it as a basis for income-targeted intervention [Page 17, lines 339-341; Page 18, lines 372-373]. Revised text is here:

Discussion:

Firearm safety programs may particularly consider age- and sex-related differences in storage practices while tailoring counseling to firearm presence, access, and household circumstances.[15,34]

Findings support continued attention to firearm safety in dementia caregiving while underscoring the importance of considering household circumstances, living arrangements, and firearm access when developing counseling and prevention strategies.

Sincerely,

Attachment Submitted filename: PLOS ONE_response letter.docx

# Editor report: Decision Letter 1

**DOI:** 10.1371/journal.pone.0360412.r003

**Authors:** Modupe Akintomide

28 Sep 2026

Unsafe firearm storage among family caregivers of older persons living with dementia

PONE-D-25-62908R1

Dear Dr. Liang,

We are pleased to inform you that your manuscript has been judged scientifically suitable for publication and will be formally accepted for publication once it meets all outstanding technical requirements.

Within one week, you will receive an e-mail detailing the required amendments. When these have been addressed, you’ll receive a formal acceptance letter and your manuscript will be scheduled for publication.

An invoice will be generated when your article is formally accepted. Please note, if your institution has a publishing partnership with PLOS and your article meets the relevant criteria, all or part of your publication costs will be covered. Please make sure your user information is up-to-date by logging into Editorial Manager at Editorial Manager® and clicking the ‘Update My Information' link at the top of the page. For questions related to billing, please contact billing support.

If your institution or institutions have a press office, please notify them about your upcoming paper to help maximize its impact. If they’ll be preparing press materials, please inform our press team as soon as possible -- no later than 48 hours after receiving the formal acceptance. Your manuscript will remain under strict press embargo until 2 pm Eastern Time on the date of publication. For more information, please contact onepress@plos.org.

Kind regards,

Modupe Akintomide

Academic Editor

PLOS One

# Editor report: Acceptance letter

**DOI:** 10.1371/journal.pone.0360412.r004

**Authors:** Modupe Akintomide

PONE-D-25-62908R1

PLOS One

Dear Dr. Liang,

I'm pleased to inform you that your manuscript has been deemed suitable for publication in PLOS One. Congratulations! Your manuscript is now being handed over to our production team.

At this stage, our production department will prepare your paper for publication. This includes ensuring the following:

* All references, tables, and figures are properly cited

* All relevant supporting information is included in the manuscript submission,

* There are no issues that prevent the paper from being properly typeset

You will receive further instructions from the production team, including instructions on how to review your proof when it is ready. Please keep in mind that we are working through a large volume of accepted articles, so please give us a few days to review your paper and let you know the next and final steps.

Lastly, if your institution or institutions have a press office, please let them know about your upcoming paper now to help maximize its impact. If they'll be preparing press materials, please inform our press team within the next 48 hours. Your manuscript will remain under strict press embargo until 2 pm Eastern Time on the date of publication. For more information, please contact onepress@plos.org.

You will receive an invoice from PLOS for your publication fee after your manuscript has reached the completed accept phase. If you receive an email requesting payment before acceptance or for any other service, this may be a phishing scheme. Learn how to identify phishing emails and protect your accounts at https://explore.plos.org/phishing.

If we can help with anything else, please email us at customercare@plos.org.

Thank you for submitting your work to PLOS One and supporting open access.

Kind regards,

PLOS One Editorial Office Staff

on behalf of

Dr. Modupe Akintomide

Academic Editor

PLOS One
